# Rapid Fabrication of High-Performance CaO-Based Integral Ceramic Mould by Stereolithography and Non-Aqueous Gelcasting

**DOI:** 10.3390/ma12060934

**Published:** 2019-03-21

**Authors:** Qiang Yang, Weijun Zhu, Zhongliang Lu, Dichen Li, Zhongrui Wang, Fu Wang

**Affiliations:** 1 State Key Laboratory for Manufacturing Systems Engineering, Xi’an Jiaotong University, Xi’an 710049, China; yangqiang@stu.xjtu.edu.cn (Q.Y.); wzr12370103150382@163.com (Z.W.); fuwang@xjtu.edu.cn (F.W.); 2 Collaborative Innovation Center of High-End Manufacturing Equipment, Xi’an Jiaotong University, Xi’an 710049, China

**Keywords:** CaO, integral ceramic mould, stereolithography, non-aqueous, gelcasting

## Abstract

A high-performance CaO-based integral ceramic mould was fabricated for investment casting by stereolithography (SLA) and non-aqueous gelcasting. The rheology of tert-butyl alcohol (TBA)-based CaO slurries and the effect of gelation parameters on the gelation time and strength of the green body were investigated to obtain a high-quality green body of the CaO-based integral ceramic mould. Then the pre-sintering and sintering processes were optimized to avoid cracks, reduce the sintering shrinkage, and improve the strength of CaO-based ceramic mould. The results showed that the CaO-based slurry with 56 vol % solid loading and 3 wt % dispersant content exhibited high stability and good flowability. The optimized gelation parameters were determined to be a monomer content of 20 wt %, a ratio of crosslinker to monomer of 0.06, an initiator content of 1 wt % and a gelation temperature of 40 °C. A reasonable sintering regime was identified to avoid cracks and guarantee a low shrinkage of 0.6%, a room temperature bending strength of 14.12 MPa and a high temperature (1200 °C) strength of 8.22 MPa. The CaO-based integral ceramic mould fabricated in this study has many advantages including excellent thermal stability, reaction-resistance to molten active alloys, ease of dissolution, and enhanced efficiency and economy in comparison to SiO_2_ or Al_2_O_3_ ceramic moulds.

## 1. Introduction

Ceramic moulds are the most critical component in investment casting processes for the fabrication of castings with complex structures and internal cavities [1]. Currently, ceramic moulds are usually made of fused silica or alumina due to their excellent properties, such as low density, good strength, high-temperature resistance, and thermal shock resistance [2,3,4]. Nonetheless, fused silica or alumina-based cores are not suitable for the investment casting of active alloy components, such as Ti, TiAl, or magnesium alloys, due to the interaction between the molten metal and SiO_2_ or Al_2_O_3_, which results in a poor surface finish of the castings [5]. Moreover, it is extremely difficult to remove fused silica- or alumina-based cores by chemical corrosion or mechanical stripping methods, as it requires a dedicated device, leads to casting corrosion, and is time-consuming [6]. Yttria presents the most negative value for the Gibbs free energy of formation among common metallic oxides and can be used in active alloy casting [7]. However, the cost is increased sharply due to the high price of Y_2_O_3_. Therefore, it is of great importance to develop a practical ceramic mould with excellent thermal stability and collapsibility but a lower cost. In recent years, CaO has been studied as a potential ceramic core material for investment casting due to its reaction-resistance to molten active alloys [8,9], ease of dissolution [10] and similar thermal expansion coefficient to those of superalloys [11].

However, despite these advantageous properties, the application of CaO has been limited due to its susceptibility to hydration upon exposure to atmospheric moisture, which increases the difficulty of fabricating CaO ceramics. Hulse prepared CaO-based ceramic cores by hot pressing CaO powder with additives of MgO, ZrO_2_, SiO_2_ and Al_2_O_3_; the relative theoretical density of these ceramic cores was at least 70–90% [11]. Wu et al. invented a type of CaO-based ceramic core by compression moulding, which was made of rare earth oxide-coated CaO powder, plasticizer and trace amounts of mineralizer [12]. Although these scholars prepared some CaO-based ceramic cores for casting, the preparation process is complicated, requiring the fabrication of a costly metal mould and the pre-treatment of CaO powder. Tsutsumi et al. developed a dental precision casting process for a beta-type titanium alloy using a mould made of electrically fused calcia particles [13]. However, this type of mould is not suitable for casting large-sized parts due to the complexity and high cost of the preparation process of electrically fused calcia. Recently, Liu et al. proposed an aqueous gelcasting method to fabricate CaO-based ceramic cores using Ca(OH)_2_ powder as the raw material and epoxy resin as the binder [10], however, the excessive sintering shrinkage of cores cannot meet the requirements of casting. With the development of additive manufacturing (AM), 3D printing technology can be employed to manufacture the ceramic mould with complex structures [14]. Zhao et al. prepared a complex-shaped CaO-based ceramic core by 3D printing using CaO powder as a precursor material, and a nano zirconia-absolute ethyl alcohol solution suspension as a binder [15]. However, the low relative density and poor mechanical properties are remaining problems that restrict the application of CaO-based ceramic cores.

An integral ceramic mould fabricated by SLA and gelcasting provides a novel approach for investment casting [16,17]. A resin mould fabricated by SLA, which has advantages of high dimensional accuracy, good rigidity, and nice surface finish, is employed to replace metal moulds or wax patterns [18]. Gelcasting is an attractive near-net shape forming technique for fabricating high-performance ceramic products with complex shapes by in-situ solidification [19,20]. In theory, it is inappropriate to fabricate CaO-based ceramic parts by aqueous gelcasting because CaO will undergo violent hydration during the preparation of the aqueous gelcasting slurries. Non-aqueous gelcasting has been successfully employed in the preparation of ceramic products, especially ceramic compositions that contain some easily hydrolytic material, such as MgO, La_2_O_3_ or AlN [21,22,23]. Therefore, the non-aqueous gelcasting process can be employed to form the CaO-based ceramic body to avoid hydration during the preparation process. Tert-butyl alcohol (TBA) has been selected as the solvent due to its low surface tension and high saturation vapor pressure, and the green body can be dried easily and with little shrinkage [24,25]. Unlike acrylamide (AM) has neurotoxic, N, N-dimethyl acrylamide (DMAA) is a TBA-soluble, low-toxicity reagent, and has been widely applied in gelcasting to replace the AM-MBAM system [26,27]. Zhang et al. prepared ZTA ceramics using DMAA as a monomer in a gelcasting system, and the flexural strength of the green bodies was as high as 21 MPa [28]. The DMAA system shows good performance in the forming process and a high-quality green body.

In this study, a rapid fabrication process for CaO-based integral ceramic moulds was developed using SLA and TBA-based gelcasting. The rheology of TBA-based CaO slurries with a high solid loading and low viscosity was investigated. The gelcasting process was optimized to obtain a homogeneous and high-strength green body. The pre-sintering and sintering processes were investigated to decrease the sintering shrinkage. Compared with the traditional injection moulding process, the proposed fabrication process is rapid and economical, and effectively solves the problem of the hydrolysis of CaO in the preparation process.

## 2. Experimental Procedure

### 2.1. Raw Materials and Reagents

**Figure 1:**
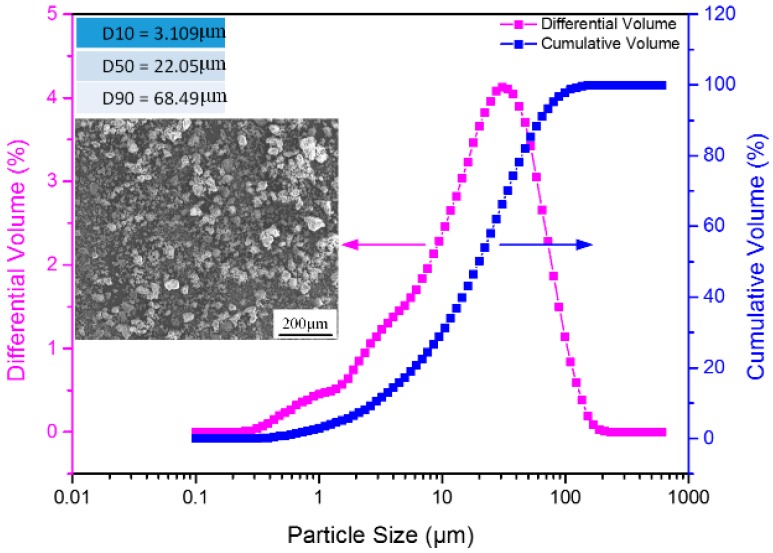
Morphology and particle size distribution of the CaO powder.

A commercial-grade CaO powder (purity ≥ 98%, Xing Tai Special Ceramics Co. Ltd., Xi’an, China) with a mean particle diameter of 22.05 μm and a BET surface area of 1.07 m^2^/g was used as the raw material. The chemical composition of the CaO powder was determined by X-ray fluorescence, and is provided in Table 1. ZrO_2_ (AR, Sinopharm Chemical Reagent Ltd., Shanghai, China) with a mean particle diameter of 1 μm was used as a sintering aid, which can reduce the sintering temperature and improve the hydrolysis-resistance of the sintered CaO-based mould due to the formation of CaZrO_3_. The morphology and particle size distribution of the CaO powder are shown in Figure 1.

### 2.2. Fabrication of CaO-Based Integral Ceramic Mould

**Figure 2:**
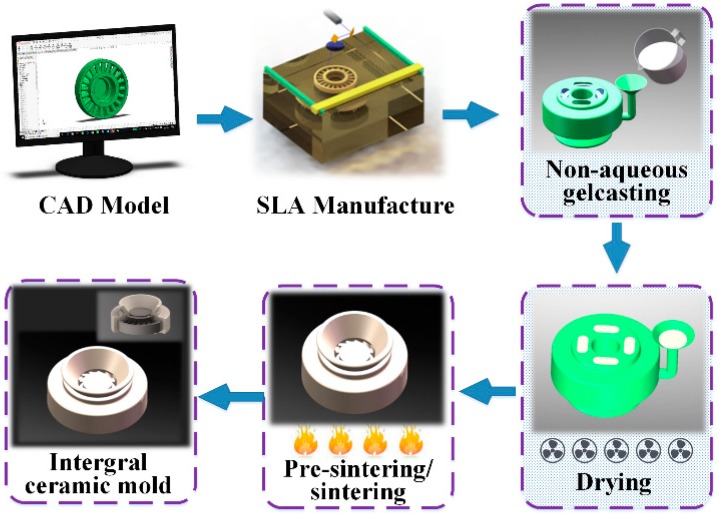
Schematic diagram of the integral ceramic mould manufacturing process.

The premixed solution was prepared by dissolving N, N-dimethylacrylamide (DMAA) and N, N-methylene diacrylamide (MBAM) in a 12:1 ratio and 3 wt % polyvinyl pyrrolidone (PVP K30) with respect to the CaO powder in the mixed solvent. An appropriate amount of CaO powder was added into the premixed solution by mechanical stirring, followed by ball milling for 40 min. The well-dispersed slurry was degassed to eliminate air bubbles. N, N-Dimethylaniline (DMA) was used as the catalyst, and 1 g of benzoyl peroxide (BPO) dissolved in 4 mL N, N-dimethylacetamide (DMAC) was used as the initiator. All the chemical reagents are of AR purity and provided by Sinopharm Chemical Reagent Ltd., Shanghai, China. The slurry with the appropriate amounts of catalyst and initiator was poured into a resin mould fabricated by an SPS600B Rapid Prototyping Machine (Shaanxi Hengtong Intelligent Machine Co., Ltd., Xi’an, China) under vacuum and vibratory conditions. The green body was subsequently placed into a vacuum freeze-dryer (Beijing Songyuan Huaxing Technology Development Co., Ltd., Beijing, China) with a freezing temperature of −40 °C, shelf temperature of 0 °C, and pressure of 10 Pa for 48 h. Finally, an integral CaO-based ceramic mould was obtained after sintering at 1400 °C for 3 h. A schematic diagram of the CaO-based integral ceramic mould manufacturing process is shown in Figure 2.

### 2.3. Characterization and Testing

Particle size analysis was conducted using a laser diffraction particle size analyser (BT-9300S, Bettersize Instruments Ltd, Dandong, China). The rheology of CaO-based slurries was characterized using a rotational rheometer (MCR302, Anton Paar GmbH, Graz, Austria). The viscosities were measured at shear rates varying from 20–200 s^−1^. A TG-DSC experiment was carried out by a TG-DSC Simultaneous Thermal Analyzer (TGA/DSC 3+, METTLER TOLEDO, Zurich, Switzerland) to determine the decomposition temperature of the gel in the green body. A scanning electron microscope (SEM, Hitachi SU-8010, Tokyo, Japan) was used to observe the microstructure of CaO powder and the fracture surface of the green and sintered bodies. The phase composition was identified by X-ray diffraction (XRD) (X’Pert Protype, PANalytical BV, Almelo, The Netherlands). The bending strength of ceramic samples (nominal size of 4 mm × 10 mm × 60 mm) were tested using a three-point bending test machine (HSST-6003QP, Sinosteel Luoyang Institute of Refractories, Luoyang, China) with a span distance of 30 mm and a crosshead speed of 6 mm/min. The apparent porosity of sintered bodies were measured by immersion in kerosene under vacuum using Archimedes’ principle.

## 3. Results and Discussions

### 3.1. Rheology of CaO Slurries

In the gelcasting process, a stable slurry with high solid loading, low viscosity and good flowability is crucial for fabricating ceramic parts with complex architectures, low shrinkage, and high strength. To prepare well-dispersed slurries, it is essential to select a suitable dispersant and its proportion. In addition, the solid loading is critical to the rheology of the slurry and the quality of the green body [29]. Therefore, the rheology of slurries was investigated to determine an appropriate proportion of dispersant and solid loading.

**Figure 3:**
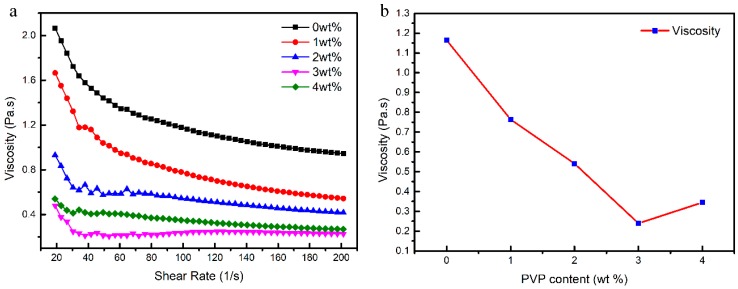
Effect of PVP content on the viscosity of 50 vol % slurries at (**a**) different shear rates and (**b**) 100 s^−1^ shear rate.

Considering its excellent solubility in TBA, film-forming property and dispersibility of CaO ceramic particles, PVP K30 was chosen as a dispersant in this system to improve the dispersion of CaO ceramic slurries. The apparent viscosity of slurries (solid loading 50 vol %) was tested to optimize the amount of dispersant, as shown in Figure 3. The slurries exhibited shear thinning behaviour and the viscosity decreased initially with an increase in the PVP content from 0 wt % to 3 wt %, after which the viscosity increased as the PVP content increased from 3 wt % to 4 wt %. It is possible that the viscosity of the ceramic slurry initially decreased due to the steric hindrance effect gradually increasing with the addition of the PVP dispersant. When the PVP content was 3 wt %, the viscosity of the slurry was the lowest, and the fluidity was optimal. However, when the adsorption of the dispersant on the surface of the particles reached saturation, if the PVP content was further increased, excess dispersant would appear in the slurry, which indicates an increase in the ionic strength of the suspension system; this causes particles agglomeration, resulting in a high-viscosity slurry. According to the above analysis, 3 wt % PVP was chosen to prepare the slurries.

**Figure 4:**
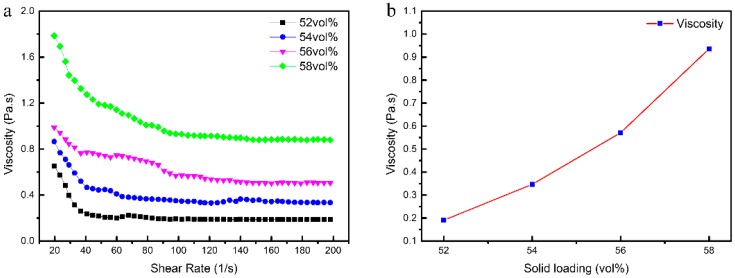
Effect of solid loading on the viscosity of CaO slurries at (**a**) different shear rates and (**b**) 100 s^−1^ shear rate.

High solid loading of a ceramic slurry plays an essential role in ensuring a sufficient density, uniformity, and strength of a green body [30]. The effect of solid loading on the viscosity of 3 wt % PVP slurries is shown in Figure 4. As shown in Figure 4a, the slurries are shear thinning and the viscosities increased with solid loading. As shown in Figure 4b, the viscosities of the slurries are less than 1 Pa·s (at a shear rate of 100 s^−1^) when the solid loading is lower than 56 vol %. In contrast, the viscosity is close to 1 Pa·s when the solid loading reaches 58 vol %, which is not suitable for gelcasting. To obtain a higher strength ceramic green body, the slurry with a solids loading of 56 vol % and a viscosity of 0.57 Pa·s was selected to form the CaO-based ceramic moulds.

### 3.2. Gelation Process of CaO-Based Slurries

The low-toxicity DMAA-MBAM gel system has been applied to fabricate SiC and ZTA; however, the gelation process of gelcasting has rarely been investigated. The effects of the monomer, ratio of crosslinker to monomer, initiator, and gelation temperature on the gelation process and resultant properties of the green bodies were studied.

**Figure 5:**
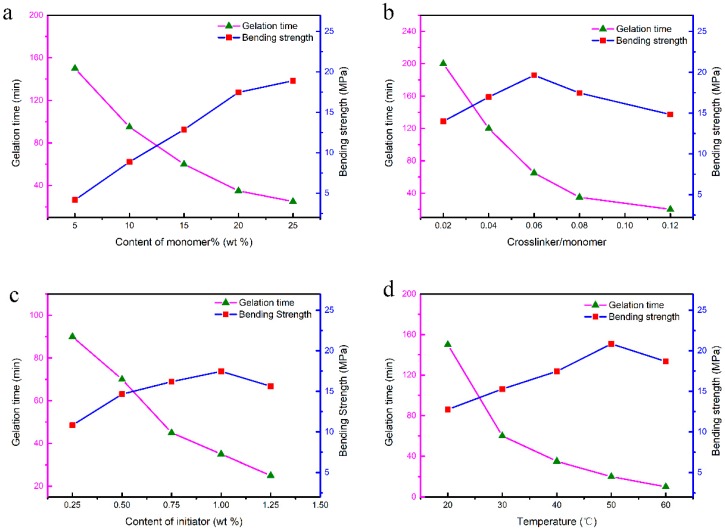
Effect of gelation parameters on gelation time and strength of green body: (**a**) content of monomer; (**b**) ratio of crosslinker to monomer; (**c**) content of initiator; (**d**) gelation temperature.

The effect of the monomer is shown in Figure 5a. The strength of the green body increased significantly with the monomer content, whereas, the gelation time showed the opposite trend. However, when the content exceeded 20 wt %, the strength improvement of the green body was not significant, and the overly rapid gelation rate is not conducive to the formation process. In addition, excess monomer also affects the homogeneity of the slurry and increases the content of organics, which will lead to a poor performance of the ceramic moulds. Therefore, a premixed solution with a monomer content of 20 wt % was used for this study.

The effect of the ratio of crosslinker to monomer on the gelation process is shown in Figure 5b. The gelation time decreased markedly with the increasing ratio of crosslinker to monomer. And the strength of the green body reached a maximum at a crosslinker/monomer ratio of 0.06. The gelation is insufficient, and the gel network is incompletely formed when the crosslinker content is low, which leads to a lower strength green body. On the other hand, the sub-chain is shorter, and the molecular weight of the gel is decreased when the crosslinker content is high, causing a decrease in the strength of the green body. According to the experimental results, the strength of the green body was highest when the ratio of the crosslinker to monomer was 0.06. Therefore, the ratio of 0.06 was selected in this study.

The effect of the content of the initiator on the gelation process is shown in Figure 5c. The gelation time decreased significantly from 90 min to 20 min with increasing initiator content. The strength of the green body increased with increasing initiator, and the maximum strength reached almost 17.4 MPa when the initiator content was 1.0 wt %. The Continued increase in the amount of initiator resulted in a slight decrease in strength. The strength of the green body was low because fewer primary radicals were formed, negatively affecting the formation of monomer free radicals, which cannot participate in the gelation process due to the insufficient addition of initiator. However, when the initiator is excessive, the slurry is prone to local polymerization and agglomeration, and the uniformity of the green body is lowered, causing a slight decrease in strength. Based on the above analysis, 1 wt % of initiator was suitable for this study.

The effect of the temperature on the gelation process is shown in Figure 5d. The figure indicates that the gelation time was significantly reduced as the temperature increased; when the temperature was raised from 20 °C to 60 °C, the gelation time was reduced from 150 min to 10 min. At the same time, the strength of the green body was also increased with increasing temperature, and the maximum strength of 20.8 MPa was reached at 50 °C. However, the strength declined slightly as the temperature continued to increase. The decomposition of BPO is an endothermic reaction. When the temperature is too low, the primary free radicals have difficulty forming, and some monomers have difficulty participating in the gelation process. When the temperature is increased, a large number of free radicals are quickly formed, and the polymerization time of the polymer is rapidly decreased. However, the non-uniform polymerization of the slurry occurred due to the rapid increase in the temperature gradient in the slurry, which led to a decrease in the strength of the green body when the temperature was too high. In addition, the resin pattern will soften, and deformation will occur at temperature higher than 50 °C, which decreases the dimensional accuracy of the ceramic mould. Hence, a temperature of 40 °C was employed for this investigation.

### 3.3. Cracks during Pre-Sintering

**Figure 6:**
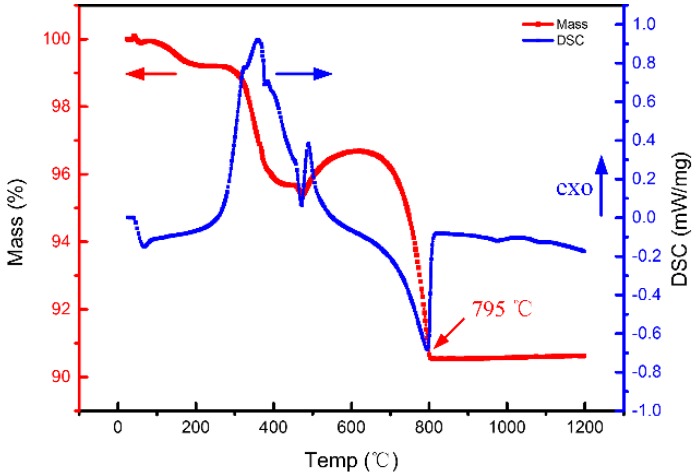
TD-DSC of green body of CaO-based ceramic mould.

The CaO-based ceramic mould is susceptible to cracking during the pre-sintering process between room temperature and 900 °C; during this step the resin mould and the organogel formed by the DMAA are gradually burned off. To study the pre-sintering characteristics of the green body of the CaO-based ceramic mould (containing the organogel), a TG-DSC analysis was carried out, and the green body was found to exhibit a mass loss of approximately 9.5% during the sintering process. The mass loss occurred in two steps; the first step occurred at approximately 450 °C, and the second step occurred at approximately 795 °C. Then, the mass remained constant, as shown in Figure 6. The mass loss in the first stage, at nearly 450 °C, is mainly caused by the decomposition and burn off of resin and organic gel. For the second stage, at nearly 795 °C, the mass loss is mainly caused by the decomposition of CaCO_3_, which is caused by a small quantity of CaO powder reacted with CO_2_ in the air during the temperature range of 500–700 °C in the previous experimental process.

**Figure 7:**
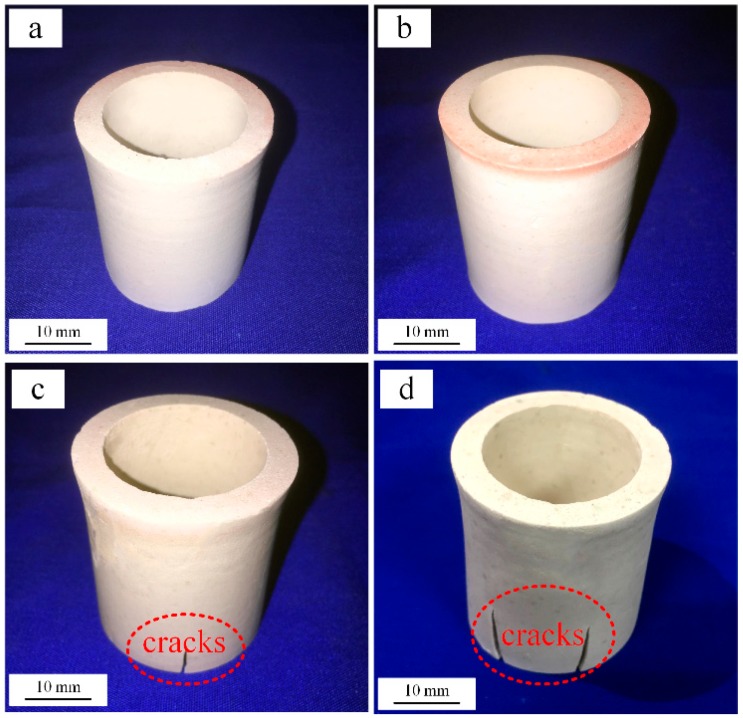
Images of CaO-based parts with different pre-sintering heating rates: (**a**) 0.5 °C/min, (**b**) 1.0 °C/min, (**c**) 1.5 °C/min, and (**d**) 2.0 °C/min.

To avoid the cracking of the ceramic mould caused by the large thermal stresses associated with uneven green body heating, an appropriate heating rate is required in the pre-sintering process, especially in the temperature range below 800 °C, in which the organic components in the green body are decomposed and burnt off. To determine the appropriate pre-sintering heating rate, CaO-based crucible parts were fabricated for experimental verification. CaO-based ceramic crucibles pre-sintered at different heating rates are shown in Figure 7. There were no cracks in the CaO-based ceramic crucibles when the heating rate was 0.5 °C/min or 1 °C/min, whereas there were obvious cracks in the crucibles when the heating rate was greater than 1.5 °C/min. Although the ceramic mould did not crack with a heating rate of 0.5 °C /min, the heating rate is too low to facilitate efficient and economical production rates. To balance the quality, efficiency and energy consumption of manufacturing, 1.0 °C/min was considered the most suitable heating rate for pre-sintering the CaO-based ceramic moulds.

### 3.4. Sintering Shrinkage and Strength of CaO-Based Mould

**Figure 8:**
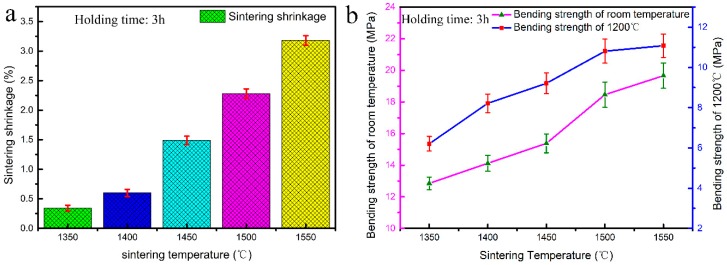
The effect of the sintering temperature on the performance of mould: (**a**) sintering shrinkage; (**b**) bending strengths at room temperature and 1200 °C.

The sintering shrinkage and bulk density are predominantly dependent on the sintering temperature and associated hold time, in case the composition of ceramic mould is pre-determined. The effect of the sintering temperature on the sintering shrinkage and properties of the CaO-based ceramic mould was investigated, as shown in Figure 8. The sintering shrinkage increased from 0.29% to 3.11% as the sintering temperature increased from 1350 °C to 1550 °C, and the bending strength, measured at room temperature and high temperature (1200 °C), increased with the increasing sintering temperature. When the sintering temperature was higher, both the room-temperature and high-temperature bending strengths were higher, and the sintering shrinkage increased. In general, the ceramic moulds used for precision casting should have a sintering shrinkage of less than 1% and a high-temperature bending strength greater than 8 MPa. According to this principle, only the sintering temperature of 1400 °C can meet these requirements.

**Figure 9:**
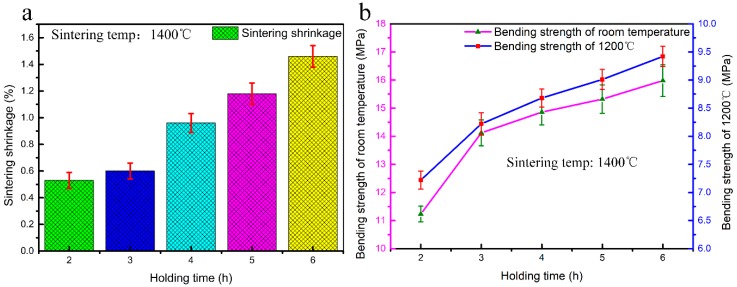
The effect of the holding time on the performance of the mould: (**a**) sintering shrinkage; (**b**) bending strengths at room temperature and 1200 °C.

The sintering holding time, in addition to the sintering temperature, is another critical factor in determining the properties of the ceramic mould. Hence, the effects of the holding time on the sintering shrinkage and properties of the CaO-based ceramic mould were also investigated, as shown in Figure 9. When the sintering temperature was 1400 °C, an increased sintering holding time (from 2 h to 6 h) resulted in a sintering shrinkage increase from 0.53% to 1.46% and increased room temperature and high temperature (1200 °C) bending strength. When the holding time is longer, the bending strength is higher, regardless of whether it was measured at room temperature or high temperature, but the sintering shrinkage is also more significant. To satisfy the requirements of precision casting, a holding time of 3 h is the most favourable for obtaining a CaO-based ceramic mould with a shrinkage of 0.6% and a high-temperature strength of 8.22 MPa.

**Figure 10:**
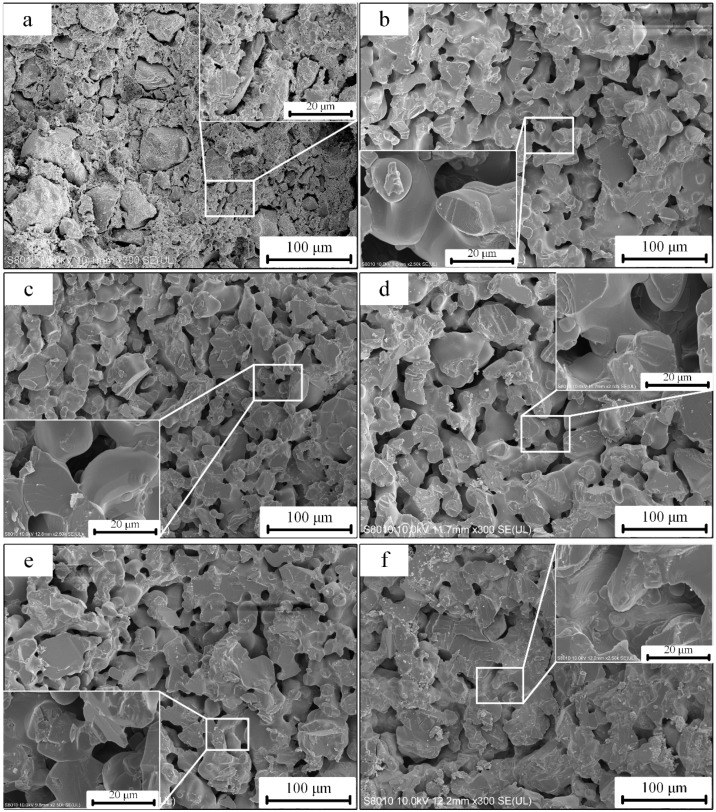
SEM of the fracture surface of green body and sintered samples with different sintering temperatures: (**a**) green body, (**b**) 1350 °C, (**c**) 1400 °C, (**d**) 1450 °C, (**e**) 1500 °C, (**f**) 1550 °C.

SEM of the fracture surfaces of the CaO-based samples with different sintering temperatures are shown in Figure 10. The microstructure of the CaO-based samples indicates enhanced densification as the sintering temperature is increased. When the sintering temperature was relatively low (1350 °C), the sample was not well sintered, and the microstructure exhibited a porous structure with little evidence of particle necking, as shown in Figure 10b, the sample exhibited a low sintering shrinkage and low strength. At a sintering temperature of 1400 °C, the sample exhibited a porous microstructure, though a large number of sintering necks formed between the CaO particles, indicating that the sample has undergone sintering but not significant densification, as shown in Figure 10c. Therefore, the sample sintered at 1400 °C had a higher strength and a lower shrinkage than the sample sintered at 1350 °C. However, when the sintering temperature was higher than 1450 °C, as shown in Figure 10d–f, the CaO grains gradually grow up, and the microstructures are relatively dense, especially for the sample sintered at 1550 °C. This result explains why the samples have good strength and large sintering shrinkage. It can also be seen from the above analysis that it is reasonable to select 1400 °C as the sintering temperature.

**Figure 11:**
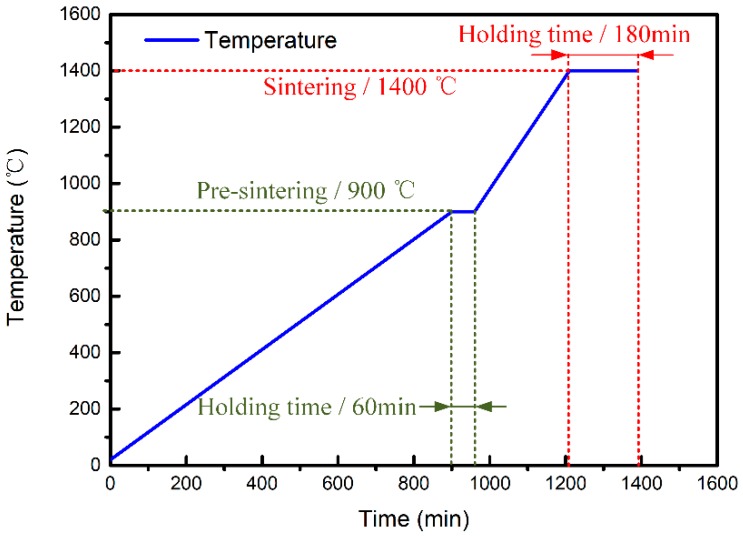
Temperature-time curve for pre-sintering and sintering processes.

An optimal sintering regime (including pre-sintering) was determined, as shown in Figure 11. In the pre-sintering stage (room temperature to 900 °C), the heating rate was 1.0 °C/min. A hold time of 60 min at 900 °C allowed for full decomposition of the organic gel (or other impurities) in the green body of the CaO-based mould. Then, the sintering temperature was raised to 1400 °C at a heating rate of 2 °C/min and held at this temperature for 3 h.

## 4. Case Study

**Figure 12:**
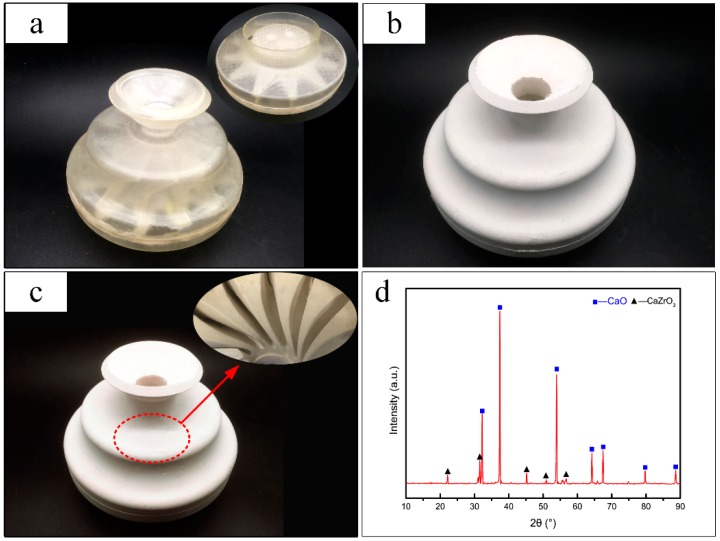
Fabrication of a CaO-based integral ceramic impeller mould and the XRD pattern of the mould: (**a**) SLA mould of impeller, (**b**) dried CaO-based ceramic body of impeller, (**c**) sintered CaO-based ceramic mould of impeller, (**d**) XRD pattern of the CaO-based sintered ceramic mould.

The CaO-based integral ceramic mould of an impeller was fabricated using the previously mentioned fabrication process, as shown in Figure 12. First, a resin mould of the impeller was fabricated by SLA, as shown in Figure 12a. Then, the CaO slurry with high solid loading and low viscosity was poured into the resin mould, and the wet green body of the ceramic mould was obtained when the ceramic slurry solidified in situ via organogel gelation. The green body was subsequently dried in a vacuum freeze dryer for 48 h, as shown in Figure 12b. Finally, the integral ceramic mould of the impeller with a complete internal structure was obtained by sintering at 1400 °C in an atmospheric sintering furnace, as shown in Figure 12c. XRD analysis was carried out to identify the phase composition of the sintered mould, as shown in Figure 12d. As seen from the XRD patterns, the mould is mainly composed of CaO and a small amount of calcium zirconate (CaZrO_3_), which is formed by the reaction of CaO and ZrO_2_ at high temperature.

Table 2 compares the bending strength, sintering shrinkage and apparent porosity for the following three materials: CaO-based integral ceramic moulds (per this study), Al_2_O_3_-based AC-1 ceramic cores (made by Beijing Institute of Aeronautical Materials, Beijing, China, and applied in the fabrication of turbine blades) and CaO-based cores (made by Huazhong University of Science and Technology, Wuhan, China). Compared with the AC-1 ceramic cores, the CaO-based integral ceramic mould has a higher room temperature and high temperature (1200 °C) bending strengths and exhibits less shrinkage. Additionally, the CaO-based integral ceramic mould has high and stable dimensional precision compared to the CaO-based ceramic cores, though its bending strength is slightly lower (but still sufficient) than that of the cores. The apparent porosity of this three materials is almost approximate. As such, it is predicted that properties of CaO-based integral ceramic moulds can meet the requirements for investment casting.

## 5. Conclusions

A rapid fabrication process to produce CaO-based ceramic moulds via SLA and non-aqueous gelcasting has been developed. The low-toxicity DMAA-MBAM gel system was successfully employed to fabricate the CaO-based integral ceramic mould with TBA solvent, which can effectively negate the hydrolysis of calcia in the preparation process. The CaO-based slurry with a high solid loading of 56 vol % and a low viscosity of 0.57 Pa·s was obtained by addition of 3 wt % dispersant. The gelation parameters with a monomer content of 20 wt %, a ratio of crosslinker to monomer of 0.06, an initiator content of 1 wt % and a gelation temperature of 40 °C were optimized to guarantee a high-quality green body. An optimized pre-sintering and sintering regime was established to ensure the dimensional precision and performance of the CaO-based mould; the CaO-based ceramic mould material exhibited a relatively low shrinkage of 0.6% and a relatively high high-temperature (1200 °C) bending strength of 8.22 MPa.

Compared to the injection moulding process, the process described in this paper is more efficient for the fabrication of moulds with complex structures and cores. The fabrication process of the CaO-based ceramic mould developed in this study is ideal for the rapid manufacturing of active metal parts with complex cavities. The process is more suitable for the rapid manufacturing of single-piece or small-batch production than the mass production due to the low efficiency of SLA. The control mechanism and method of near-zero shrinkage and the casting performance of CaO-based mould are still need to be concerned in the further study.

## Figures and Tables

**Table 1:** Chemical analysis of raw CaO powder.

Chemical	CaO	MgO	SiO_2_	Al_2_O_3_	Fe_2_O_3_
wt %	98.10	0.81	0.72	0.25	0.12

**Table 2:** Properties of CaO-based integral ceramic mould, Al_2_O_3_-based AC-1 ceramic cores and CaO-based ceramic cores.

Properties	CaO-Based Integral Ceramic Moulds	Al_2_O_3_-Based AC-1 Ceramic Cores [31]	CaO-Based Ceramic Cores [10]
Bending strength at room temperature/MPa	14.12 ± 0.28	9–12	25 ± 1.2
Bending strength at high temperature (1200 °C)/MPa	8.22 ± 0.26	0.8–1.6	Not mentioned
Sintering shrinkage/%	0.6 ± 0.06	1.5	11.31
Apparent porosity/%	32.8 ± 1.2	34	30.5
